# Supplementary material for: A dual-interface load transfer model for precast concrete-cored cemented soil piles considering progressive damage
Source: PLoS One. 2026 Jun 1;21(6):e0349876. doi: 10.1371/journal.pone.0349876 (PMC13225360; doi:10.1371/journal.pone.0349876)
Supplement: Attachment — Submitted filename: Response_to_Reviewers.docx [file pone.0349876.s004.docx]

Dear Editor and Reviewers,

Thank you for your decision letter regarding our manuscript (PONE-D-26-16041). We sincerely thank the editorial personnel and the reviewers for their professional review work, constructive comments, and valuable suggestions on our article.

The following changes have been made in response to the journal requirements:

1. The copyright issue of Figure 1 has been resolved (replaced with an original schematic diagram).

2. The funding statement and funder role have been amended in the cover letter.

3. The data availability statement has been updated.

4. The permit information has been added to the Methods section (Section 4.1), stating that no specific permits were required for this study.

5. In response to Reviewer #3's comment, Figs 8 and 10 have been revised. The updated figures have been uploaded.

We sincerely thank the reviewers for their valuable comments, which have helped make our manuscript more rigorous. In the following section, we provide our point-by-point responses to the reviewers' comments.

Sincerely,

Yating Zhu and co-authors

**Reviewer #1:**

**Comment 1:** The introduction lacks sufficient clarity in identifying the specific research gaps. The manuscript should clearly state the key differences between the proposed “coupled exponential damage model and elastoplastic model” and existing PCCS load-transfer models in the literature, and discuss relevant pioneering work accordingly. It should also explicitly point out the limitations of previous models in characterizing interface damage and dual-interface coupling mechanisms, as well as the specific approaches adopted in this study to overcome these limitations.

**Response 1:** Thank you very much for your valuable suggestion. We have revised the introduction to further clarify the research gap, with a focus on elaborating the limitations of existing load transfer models for precast concrete-cored cemented soil (PCCS) piles: most existing models adopt a single-interface assumption, which fails to capture the realistic interaction among the precast concrete core, cemented soil, and surrounding soil; moreover, the interface constitutive models are overly simplified and cannot accurately describe the progressive damage and nonlinear softening characteristics of the dual interfaces under loading. On this basis, we further elucidate the key differences between our proposed dual-interface coupled model and existing models, and explicitly state that by introducing a coupled exponential damage model and elastoplastic model, this study overcomes the aforementioned limitations, thereby making the research motivation and innovations clearer.

**Comment 2:** The current reference list is dominated by citations from Chinese scholars. A more diverse range of international references, including those from international academic conferences, should be incorporated to reflect the latest global progress in PCCS, composite piles, and interface damage modeling, thereby enhancing the international perspective and academic relevance of the study.

**Response 2:** Thank you very much for your suggestion. From the perspectives of the dual-interface coupling mechanism of PCCS piles and interface nonlinear softening, we have added two international references [22] and [23] in the introduction. These two references propose a generalized nonlinear softening load transfer model for axially loaded piles and a generalized elastoplastic model considering modulus degradation and skin friction softening, respectively. By incorporating the above references, we have enriched the research perspective on interface damage and load transfer theory in this paper, enhanced the international visibility of the study, and improved the imbalance between domestic and international references in the original manuscript.

**Comment 3:** The decomposition expression for pile-head settlement in Equation (1) $\text{S}\left( \text{z} \right)\text{ =}{{\text{ }\text{s}}_{\text{pc}}\text{ + }\text{s}}_{\text{cs}}\text{ + }\text{S}_{\text{s}}$ has been adopted in several previous studies. It is suggested that a proper citation be added to clarify the origin of this expression and strengthen the rigor of the theoretical derivation.

**Response 3:** Thank you very much for your suggestion. We have added a citation [31] to Equation (1) in Section 2.1.

**Comment 4:** The manuscript employs a variety of symbols that are critical to the model description. A comprehensive table of symbols and parameters is recommended, listing the name, physical meaning, and unit of each symbol and parameter to improve readability.

**Response 4:** Thank you very much for your suggestion. We have added a table of Nomenclature, which is placed after the Conclusions section and before the References section.

**Comment 5:** The specimen details in the interface shear tests are relatively brief. Additional experimental information, such as the curing conditions of specimens, may be supplemented.

**Response 5:** Thank you very much for your suggestion. We have supplemented the curing conditions in Section 4.2. In the interface shear test section for the precast concrete core–cemented soil interface, we have added the moisture curing condition. In the interface shear test section for the cemented soil–surrounding soil interface, we have also added the moisture curing condition for the specimens. These additions make the experimental details more complete and rigorous.

**Comment 6:** The mechanism analysis of axial force along the pile shaft and shaft friction lacks sufficient depth. The manuscript only reports the observed “C-shaped distribution” of axial force along depth but does not explain the underlying causes, such as the stiffness difference between the PC core and cemented soil, the non-uniform mobilization of shear stress at the two interfaces, and the variation in the proportion of pile-tip resistance. A brief mechanistic explanation is suggested.

**Response 6:** Thank you very much for your suggestion. We have deepened the mechanistic analysis of the axial force and shaft resistance along the pile, and have supplemented the explanation of the formation mechanism of the C-shaped distribution in the text (Section 5.2.1). The C-shaped distribution of axial force in the cemented soil arises from depth-dependent variations in load transfer: interface slip and negative skin friction in the shallow section reduce the axial force of the cemented soil, while increased confining pressure and shear hardening at greater depths mobilize positive skin friction, thereby enhancing its bearing capacity.

**Reviewer #2:**

**Comment 1:** Lines 69-70: Some claims are too strong. It states that the iterative algorithm has “guaranteed convergence.” The “guaranteed convergence” should be revised unless a formal proof or additional convergence study is provided.

**Response 1:** Thank you very much for your suggestion. Regarding the overly absolute statement, we have revised the original sentence in the introduction from "An efficient iterative solution algorithm with guaranteed convergence is developed to compute the mechanical response under axial loading" to a more rigorous expression: "An iterative calculation method with convergence characteristics is established to solve the mechanical response under axial loading."

**Comment 2:** The assumptions, considering the pile, cemented soil and surrounding soil as homogeneous, isotropic, and linearly elastic materials, are acceptable for a simplified analytical model. However, the authors should briefly discuss the limitation of this assumption and clarify the scope of applicability more explicitly.

**Response 2:** Thank you very much for your suggestion. We have supplemented the discussion on the limitations of the simplified analytical model assumptions after the basic assumptions section (Section 2.2). Treating the PC pile, cemented soil, and surrounding soil as linearly elastic materials is a common simplification in analytical models of composite piles (see references [28] and [29]). This idealization facilitates theoretical derivation and improves computational efficiency, but it neglects the heterogeneity, anisotropy, and nonlinear characteristics of natural soils. Therefore, the proposed model is suitable for preliminary design and engineering estimation under small deformation conditions.

**Comment 3:** Line 249 and Line 271: The determination of the critical relative displacements $\text{S}_{\text{u}\text{1}}$ and $\text{S}_{\text{u}\text{2}}$ could be clarified further. Since the interface test curves may vary slightly under different test conditions, the authors are encouraged to explain how these representative values were determined. Please clarify whether they correspond to peak-displacement values, fitted transition points, or averaged values from multiple tests.

**Response 3:** Thank you very much for your suggestion. We have supplemented the explanation in the text (Section 4.2) regarding the determination of $\text{S}_{\text{u}\text{1}}$ and $\text{S}_{\text{u}\text{2}}$, which are taken as the peak values of the interface shear curves. Multiple sets of interface direct shear tests under different working conditions were conducted according to actual engineering conditions. The skin friction and corresponding displacement at the peak point for each working condition were extracted, and their average values were taken as the representative values, from which $\text{S}_{\text{u}\text{1}}$, $\text{S}_{\text{u}\text{2}}$, $\text{τ}_{\text{u}\text{1}}$ and $\text{τ}_{\text{u}\text{2}}$ were ultimately determined.

**Comment 4:** The Introduction could be improved by discussing some closely related studies. I suggest the authors cite and discuss the following relevant paper:

Li, L., Lai, N., Zhao, X., Zhu, T., & Su, Z. (2023). A generalized elastoplastic load-transfer model for axially loaded piles in clay: Incorporation of modulus degradation and skin friction softening. Computers and Geotechnics, 161, 105594.

Ni, P., Song, L., Mei, G., & Zhao, Y. (2017). Generalized nonlinear softening load-transfer model for axially loaded piles. International Journal of Geomechanics, 17(8), 04017019.

**Response 4:** Thank you very much for your suggestion. We have cited and discussed the two papers recommended by the reviewer as references [22] and [23] in the introduction. These two references propose load transfer models considering nonlinear softening and modulus degradation for axially loaded piles, respectively, which enrich the existing research on load transfer models incorporating interface softening characteristics. The relevant findings have been reviewed and summarized in the research background of this paper.

**Comment 5:** Line 92: For clarity, please add explicit definitions of all symbols in Eq. (1), including $\text{S}\left( \text{z} \right)\text{ }$, $\text{s}_{\text{pc}}$, $\text{s}_{\text{cs}}$, and $\text{S}_{\text{s}}$.

**Response 5:** Thank you very much for your suggestion. We have added the definitions of all symbols in Equation (1) below the equation in Section 2.1.

**Comment 6:** Lines 152-153: The node numbering scheme is somewhat unconventional, since Node 1 is defined at the pile toe and Node m+1 at the pile head. Please add a brief explanatory note in the text or figure so that readers can follow the recursion procedure more easily.

**Response 6:** Thank you very much for your suggestion. We have supplemented the explanation of the recursive calculation procedure in Section 3.2. The pile is divided into m equal-length elements, generating a total of m+1 nodes. Among these, Node 1 is located at the pile toe and Node m+1 at the pile head. The recursive analysis proceeds upward from the pile toe, because the displacement at the pile toe is generally the smallest along the entire pile shaft. In this model, the analysis is initiated with a specified pile toe displacement as the boundary condition. Performing the recursive calculation upward from the pile toe minimizes the error introduced by the assumed displacement boundary condition, thereby more accurately reflecting the actual mechanical response of the pile.

**Comment 7:** Lines 265–266: There is an obvious editing error: “Error! Reference source not found.” This should be corrected.

**Response 7:** Thank you very much for your suggestion. We have corrected the citation error that appeared in the manuscript and carefully proofread all citations throughout the paper.

**Reviewer #3:**

**Comment 1:** The PC pile, cemented soil, and surrounding soil are assumed to be linearly elastic. In reality, plastic deformation and damage occur in cemented soil and surrounding soil under loading. The authors are suggested to explain the rationale for this modeling assumption and indicate whether relevant analyses have been conducted.

**Response 1:** Thank you very much for your suggestion. We have supplemented the analysis and discussion of the limitations of the relevant simplifying assumptions in the "Basic Assumptions" section (Section 2.2). Simplifying the PC pile, cemented soil, and surrounding soil as linearly elastic materials highlights the nonlinear mechanical behavior of the interfaces and ensures computational efficiency and engineering practicality of the model. This simplification is also a common approach in analytical models of composite piles (references [28] and [29]). Although this idealization facilitates theoretical derivation, it neglects the heterogeneity, anisotropy, and nonlinear characteristics of natural soils. Therefore, the model is more suitable for preliminary design and engineering estimation under small deformation conditions.

**Comment 2:** The specific value of the convergence criterion used in the iterative algorithm is not provided. Additionally, the authors should explain whether the algorithm remains convergent for layered soil conditions.

**Response 2:** Thank you very much for your suggestion. We have supplemented the specific value of the convergence criterion in the text (Section 3.3) and explained that the algorithm remains convergent under layered soil conditions. The iterative calculation in this paper adopts a relative error convergence criterion, with the convergence tolerance controlled within 1% to 5%, which meets the general requirements for engineering analysis and design. The algorithm is also applicable to layered soils; it only requires updating the corresponding soil parameters according to the layer thickness and continuing the iteration. Since the engineering case in this paper primarily involves a sandy soil foundation, the calculation example uses a single-layer soil for analysis.

**Comment 3:** Only the final values of the exponential damage model parameters for the inner interface ( ) are reported. The fitting curves and residuals are not presented, making it impossible to assess the reliability of the calibration.

**Response 3:** Thank you very much for your suggestion. We have supplemented the fitting curves (Figs 8 and 10) in the revised manuscript, and added the residual analysis results in Sections 4.2.1 and 4.2.2. To evaluate the reliability of the parameter calibration, we provide the residual analysis results below. For the PC pile–cemented soil interface (exponential damage model), the R² values for the three datasets are 0.987, 0.990, and 0.998, with RMSE values of 7.32, 17.57, and 10.07 kPa, and mean residual values of +3.68, -13.75, and +4.46 kPa, respectively. For the cemented soil–surrounding soil interface (elastic-perfectly plastic model), the R² values for the four datasets are 0.952, 0.983, 0.990, and 0.978, with RMSE values of 4.60, 2.93, 3.10, and 4.24 kPa, and mean residual values of -2.05, -0.46, +0.79, and -0.58 kPa, respectively. The above results are the supplementary outcomes of the residual analysis.

**Comment 4:** The laboratory direct shear tests were performed on scaled models, which introduce scale effects on material strength and interface properties. The authors should clarify how the scaled parameters can be directly applied to the 14 m full-scale pile.

**Response 4:** Thank you very much for your suggestion. In this study, the 14 m full-scale pile was scaled down according to the similarity theory, with a geometric similarity ratio of 1:14 and an elastic modulus similarity constant of 1.446. Both geometric and physical-mechanical similarity relationships were satisfied. Dimensionless parameters such as strain and Poisson's ratio can be directly applied to the analysis of the full-scale pile. The peak shear stress and peak displacement at the interface are primarily controlled by the material mix proportion, interface roughness, and normal stress, and the scaled tests were consistent with the prototype engineering conditions in these respects. Due to limitations in test conditions, residual size effects may still exist in the scaled tests; however, the model calculation results are in good agreement with the field test data of the full-scale pile (R² = 0.9746), indicating that the impact on the overall bearing capacity prediction is within an acceptable range for engineering purposes. The relevant content has been supplemented in Section 4.2.1 of the manuscript.

**Comment 5:** The elastoplastic parameters for the outer interface ( ) are derived solely from sand. The authors should address whether these parameters are applicable to clay or silty clay.

**Response 5:** Thank you very much for your suggestion. The main foundation soil at the engineering prototype site on which this study is based is sandy soil. Therefore, we specifically conducted direct shear tests on the cemented soil–sand outer interface to obtain the elastoplastic interface parameters under the corresponding working conditions. For other soil conditions such as clay or silty clay, it is recommended that targeted interface shear tests be conducted according to the actual engineering conditions, or that the corresponding test parameters or empirical parameters be obtained from relevant literature, which can then be substituted into the calculation method proposed in this paper for iterative analysis. The core contribution of this study is to provide a general theoretical calculation framework that can be used to compute and analyze the vertical bearing characteristics of precast concrete-cored cemented soil piles under different working conditions by substituting the interface parameters and soil layer distribution information corresponding to those conditions. The above explanation has been supplemented in Section 4.2.2 of the revised manuscript.

**Comment 6:** The origin of the cemented soil elastic modulus is unclear. The authors must specify whether it was obtained from laboratory tests, empirical values, or literature references.

**Response 6:** Thank you very much for your suggestion. We have supplemented the source of the relevant parameters in Section 5.1 of the manuscript. In this study, the elastic modulus of the cemented soil was obtained through laboratory unconfined compression tests in accordance with the "Technical Code for Ground Treatment of Buildings" (JGJ 79-2012). The mix proportion and raw materials of the sandy cemented soil specimens were consistent with those used in a PCCS pile prototype project in Jiangxi Province. After 28 days of standard curing, the average elastic modulus measured from three sets of parallel tests was 1.458 GPa, ensuring that the parameter values were highly consistent with the prototype engineering conditions.

**Comment 7:** Only one field test pile was used in this study. The small sample size prevents validation of the model’s generalizability to different pile lengths, pile diameters, and soil conditions, and cannot eliminate random errors.

**Response 7:** Thank you very much for your suggestion. The issue of sample size you raised does exist, and this is one of the main limitations of this study. Due to field constraints, we obtained complete field test data from only one test pile. Therefore, this single test pile is primarily used to demonstrate the parameter calibration procedure and solution feasibility of the model, rather than to validate its statistical generalizability. We have explicitly acknowledged this limitation in the Conclusions section (Section 6) of the revised manuscript, and plan to further validate the model's generalizability under different pile lengths, pile diameters, and soil conditions through multi-sample, multi-condition experimental and numerical analyses in future research.

**Comment 8:** The authors are requested to carefully proofread the paper for linguistic polishing and correct all formatting errors, including incorrect citations (e.g., “Error! Reference source not found”).

**Response 8:** Thank you very much for your suggestion. We have corrected the citation errors that appeared in the manuscript and carefully proofread all citations throughout the paper. Additionally, we have thoroughly reviewed the language and formatting of the entire manuscript to ensure clarity and correctness.
